# The anticancer thiosemicarbazone triapine exerts immune-enhancing activities via immunogenic cell death induction and FAS upregulation

**DOI:** 10.1186/s40164-025-00700-0

**Published:** 2025-08-22

**Authors:** Bianca Stiller, Alessia Stefanelli, Hemma Schueffl, Marlene Mathuber, Nadiya Skorokhyd, Judith Gufler, Christine Pirker, Martin Holcmann, Rostyslav Panchuk, Maria Sibilia, Doris Marko, Walter Berger, Christian R. Kowol, Sonja Hager, Petra Heffeter

**Affiliations:** 1 Center for Cancer Research and Comprehensive Cancer Center, Medical University of Vienna, Borschkegasse 8a, Vienna, 1090 Austria; 2 Department of Food Chemistry and Toxicology, Faculty of Chemistry, University of Vienna, Waehringer Str. 42, Vienna, 1090 Austria; 3 Institute of Inorganic Chemistry, Faculty of Chemistry, University of Vienna, Waehringer Str. 42, Vienna, A-1090 Austria; 4 Department of Regulation of Cell Proliferation and Apoptosis, Institute of Cell Biology, National Academy of Science of Ukraine, Drahomanov Str., 14/16, Lviv, 79005 Ukraine; 5 Research Cluster ‘Translational Cancer Therapy Research’, University of Vienna and Medical University of Vienna, Vienna, Austria

**Keywords:** Anticancer therapy, Thiosemicarbazones, Triapine, Immunogenic cell death, Immunomodulation, Adaptive immune system, Cytotoxic t cells, FAS, NFκB

## Abstract

The anticancer thiosemicarbazone Triapine is currently in a phase III clinical trial in combination with radiation therapy and cisplatin. Noteworthy, while radiotherapy induces an immune-activating cell death, so called immunogenic cell death (ICD), cisplatin possesses immunomodulatory and ICD-enhancing functions. Interestingly, although there are several indications that suggest that Triapine could also enhance the immune recognition of cancer cells, no investigations in this direction have been reported so far. Indeed, immune cells (especially cytotoxic T-cells) were found to enhance the anticancer activity of Triapine. This effect might be based on endoplasmic reticulum (ER) stress induction, which on the one hand led to ICD of the cancer cells as indicated by ATP release, calreticulin exposure, high-mobility group box 1 secretion and in vivo vaccination experiments. On the other hand, the Triapine-induced ER stress resulted in FAS upregulation in cell culture as well as in vivo via NFκB signaling. This, in turn, rendered cancer cells more susceptible to FASL (predominantly expressed by lymphoid immune cells)-induced caspase 8-mediated apoptosis. Consequently, our study is the first to unveil the significant role of the (adaptive) immune system in the anticancer activity of Triapine, positioning it as a promising partner for combination with immunotherapy and other immunogenic agents.

**Supplementary Information:**

The online version contains supplementary material available at 10.1186/s40164-025-00700-0.

## To the Editor

Triapine is tested in combination with cisplatin and radiotherapy in a phase-III clinical trial against advanced-stage cervical and vaginal cancers (NCT02466971). Noteworthy, both combination partners strongly interact with the tumor immune microenvironment [1]. However, the immunogenicity of Triapine was not evaluated so far, despite several indications: (1) Triapine chelates iron and copper ions, which are important immune regulators [2], e.g. via nuclear factor kappa B (NFκB) [3]. (2) Triapine induces a specific form of endoplasmic reticulum (ER) stress [4], which could result in immunogenic cell death (ICD). (3) Combination of Triapine with radiotherapy and cisplatin was effective especially in cervical carcinoma, a cancer type with high neo-antigenicity [5]. Consequently, this study examined how Triapine influences the immune recognition of cancer cells and assessed its potential as an immunologically active anticancer drug.

### Triapine activity relies on the immune system and cytotoxic T-cells

**Fig. 1:**
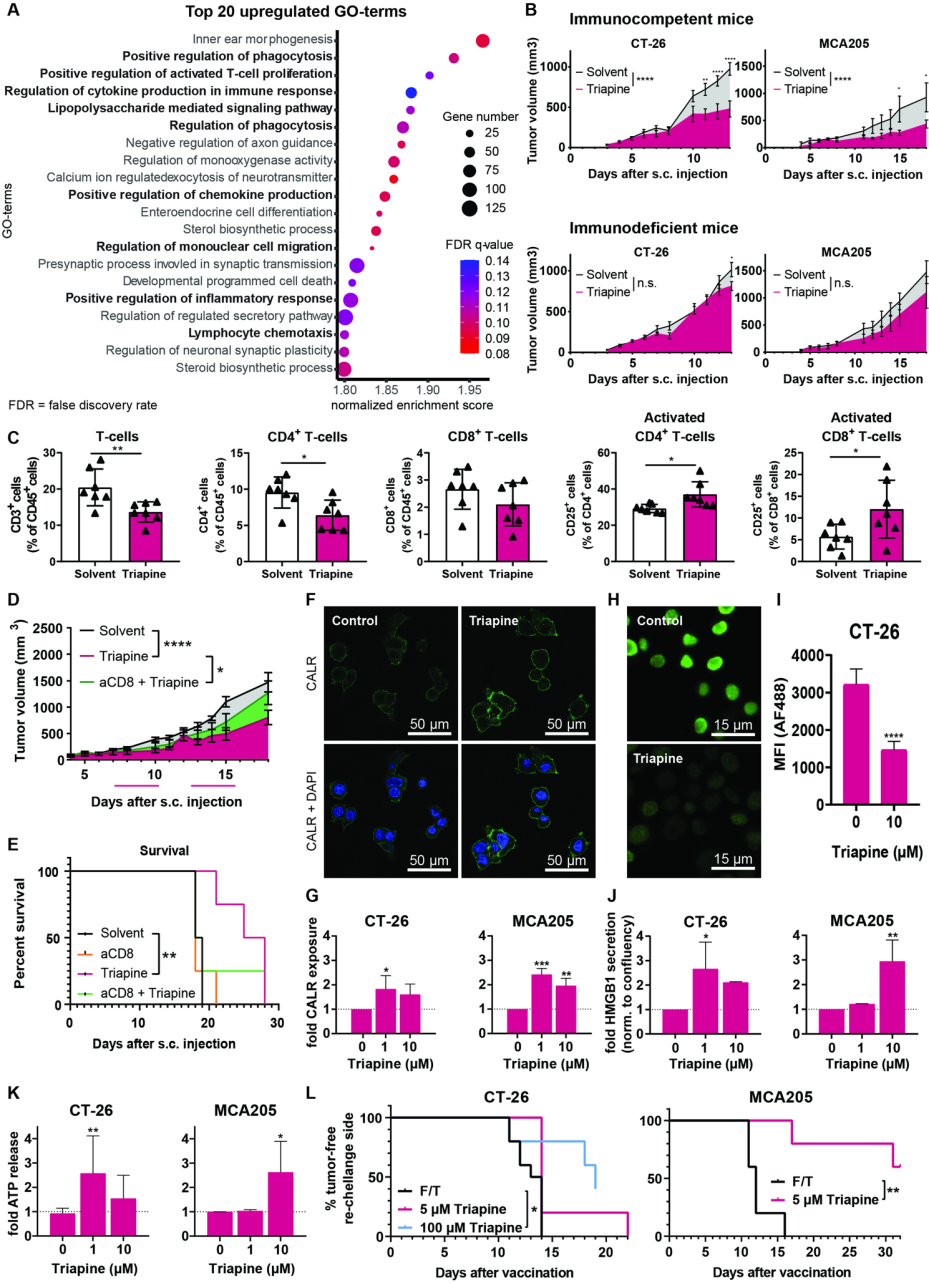
The role of the adaptive immune system in the anticancer activity of Triapine. (**A**) Top 20 upregulated GO-terms in a transcriptomic dataset (SW480, 1 µM Triapine, 15 h) compared to control. (**B**) Impact of immune status on the anticancer activity of Triapine in murine allograft models. Mean tumor volume (mm^3^) ± SEM is given. (**C**) Flow cytometry analysis on day seven of CT-26 tumor-infiltrating immune cells of female Balb/c mice. Mean percent cells of the (grand)parental gate ± SD is given. Effect of a CD8-blocking antibody on (**D**) tumor growth (mean tumor volume (mm^3^) ± SEM) of Triapine-treated CT-26 allografts and (**E**) overall survival. Pink lines indicate Triapine treatment. (**F**) Representative confocal microscopy images of CT-26 cells stained for CALR (green) and DAPI (blue) after 24 h Triapine treatment (10 µM, scale bar: 50 μm). (**G**) Flow cytometry analysis of CALR (24 h). (**H**) Representative fluorescence images and (**I**) associated quantification of CT-26 cells stained for HMGB1(green) and DAPI (blue) after 24 h Triapine treatment (10 µM; scale bar: 15 μm). Triapine induced (**J**) HMGB1 and (**K**) ATP release into the supernatant after 24 h treatment. Values given are the mean ± SD of at least three independent experiments, normalized to untreated control. (**L**) Percentage of tumor-free mice at the site of re-challenge site after vaccination with Triapine-treated cancer cells. The control group received freezed/thawed (F/T)-lysed cells. Significant differences were calculated by mixed-effects analysis corrected for multiple comparisons by Sidak, unpaired T-test, one-way, two-way ANOVA corrected for multiple comparisons by Sidak or Dunnett, or Log-rank and Mantel-Cox post-test (**** *p* < 0.0001, *** *p* < 0.001, **p < 0.01; *p < 0.05, n.s. = not significant)

Transcriptomic datat [6] of Triapine-treated human colon carcinoma SW480 cells showed that nine of the top 20 upregulated gene ontology (GO) terms were immune-related (Fig. 1A, S1A). Inspired by this, we investigated the role of the immune system on anticancer activity of Triapine in tumor-bearing mice. Indeed, while Triapine reduced tumor growth of three murine tumor models (CT-26 colon carcinoma, MCA205 fibrosarcoma, B16-F10 melanoma) in immunocompetent mice, no or only a minor effect in immunodeficient (C.B.17 SCID) mice was observed (Fig. 1B, S1B).

Subsequently, the impact on tumor-infiltrating immune cell populations in CT-26 tumors was analyzed by flow cytometry (Fig. 1C and S2A-S2B). Interestingly, while overall leukocyte and B-cell populations remained widely unchanged (Fig. S2C), T-cells were significantly decreased after 4 days of Triapine treatment (Fig. 1C). This effect was mainly based on a decline of CD4^+^ T-cells. In contrast, although the overall cytotoxic (CD8^+^) T-cell population remained unchanged, a 2.1-fold increase of activated (CD25^+^) CD8^+^ T-cells was observed. To confirm the importance of CD8^+^ T-cells for Triapine therapy, an experiment with CD8-blocking antibodies (aCD8) was performed (Fig. 1D-E and S3A-S3B). Indeed, under Triapine treatment CD8^+^ T-cell-depleted mice showed enhanced tumor growth and, thus, reduced overall survival compared to mice with functional CD8^+^ T-cells (Fig. 1D-E). No change in tumor growth or survival was observed due to CD8^+^ T-cell-depletion alone (Fig. S3C). In line, in an ex vivo experiment, CD3-stimulated splenocytes reduced the number of Triapine-pretreated CT-26 cells more efficiently compared to untreated cells (Fig. S4).

### Triapine induces ICD and a systemic adaptive immune response

A therapy-induced mechanism for CD8^+^ T-cell activation is ICD. Consequently, the three most prominent ICD hallmarks [7] were analyzed in our three murine as well as one human SW480 cancer cell line. Indeed, Fig. 1F-K, S5 confirm significant calreticulin (CALR) exposure and ATP release into the culture medium. In addition, high-mobility group box 1 (HMGB1) was translocated from nucleus and released after 24 h and 48 h treatment in murine and human cancer cells, respectively. To investigate whether Triapine induces a systemic adaptive immune response also in vivo, vaccination experiments were performed: immune-competent mice were inoculated with Triapine-treated, dying cancer cells to stimulate immune recognition, followed one week later by re-challenge with viable, untreated cells. The ideal Triapine concentration for vaccination was determined by annexin V+/propidium iodide staining (Fig. S6A-S6C). Vaccination with Triapine-treated MCA205 and CT-26 cells significantly delayed tumor formation compared to freeze/thaw (F/T)-treated negative controls (Fig. 1L, S6D). Additionally, no tumor growth at the re-challenge site was observed in 60, 50 or 40% of mice vaccinated with MCA205, B16 or CT-26 cells, respectively, indicating involvement of a systemic immune response.

### Triapine stimulates FAS upregulation via ER stress and NFκB

**Fig. 2:**
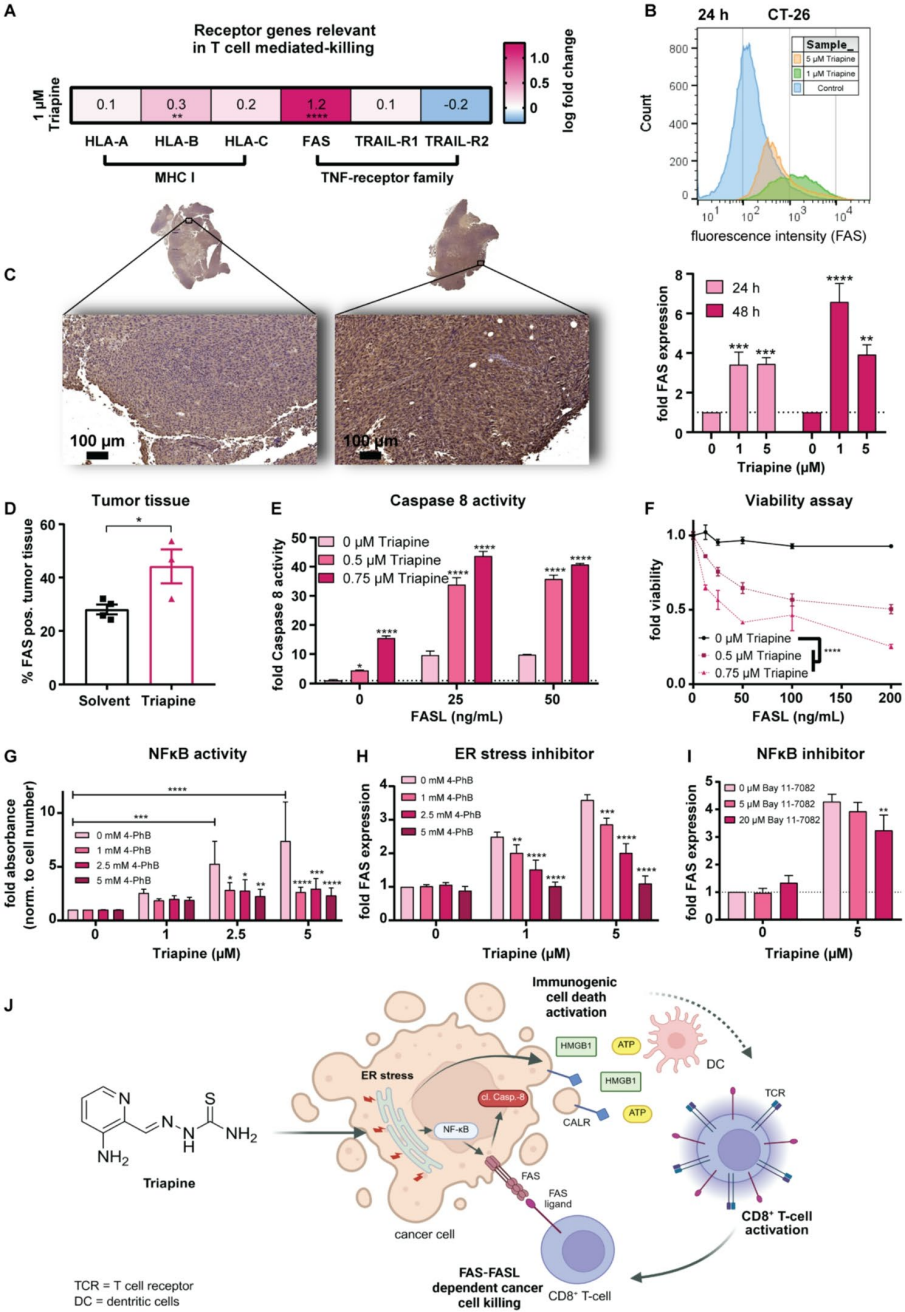
Triapine stimulates FAS upregulation via ER stress and NFκB. (**A**) Expression of receptor genes in SW480 cells (1 µM Triapine, 18 h). Values are mean log fold change compared to control. (**B**) Representative flow cytometry histograms and quantification (mean fluorescence intensities (MFI) ± SD) of FAS expression of Triapine-treated CT-26 cells. (**C**) Representative IHC images (scale bar: 100 μm) and (**D**) associated quantification of FAS expression (mean % ± SEM) in CT-26 tumors after treatment with Triapine. (**E**) Caspase 8 activity and (**F**) cell viability of HCT116 cells after 24 h Triapine preincubation, followed by 24 h FASL treatment. Values are mean ± SD from one representative experiment out of three, normalized to untreated control. (**G**) Impact of the ER-stress inhibitor 4-PhB on Triapine-induced NFκB activity was analyzed using NFκB reporter HEK293-Blue™ hTLR7 cells after 24 h. Values are mean ± SD of three independent experiments normalized to control and cell number. Impact of (**H**) 4-PhB or ± (**I**) Bay 11-7082 on Triapine-induced FAS expression in HCT116 cells with functional caspase 8 signaling after 24 h treatment. Values are MFI ± SD of three independent experiments, normalized to control. Significance was determined by one-way, two-way ANOVA with Bonferroni´s or Dunnett´s multiple comparisons test or unpaired T-test (**** *p* < 0.001, *** *p* < 0.001, ** *p* < 0.01, * *p* < 0.05). (**J**) Scheme summarizing the immune-enhancing activities of Triapine via induction of ICD and FAS upregulation.

Besides immune cell attraction, visibility of cancer cells by MHC class I or death receptors like FAS is crucial for T-cell-mediated killing [8]. In good agreement with the transcriptomic analysis (Fig. 2A), Triapine upregulated FAS in vitro (Fig. 2B, S7A-7B) and in vivo (Fig. 2C-D, S8). Accordingly, Triapine-induced FAS stimulation led to increased responsiveness toward FAS ligand (FASL), demonstrated by caspase 8 activation (Fig. 2E) and reduced cell viability (Fig. 2F). Noteworthy, FAS expression can be activated by ER stress-induced NFκB signaling [9, 10]. To test this pathway with respect to Triapine, HEK293-Blue™ NFκB reporter cells and the ER stress inhibitor 4-phenylbutyrate (4-PhB) were employed. Indeed, 4-PhB decreased Triapine-induced NFκB activation (Fig. 2G). Furthermore, Triapine-mediated FAS upregulation was reduced by 4-PhB (Fig. 2H, S7C) and the NFκB inhibitor Bay 11-7082 (Fig. 2I, S7D), indicating that Triapine-induced ER stress leads to FAS upregulation by NFκB.

In conclusion, Triapine enhanced the visibility of cancer cells to the immune system (especially to CD8^+^ T-cells) by ICD induction and FAS upregulation (Fig. 2J). Consequently, Triapine might be a promising partner for immunotherapy and immunogenic compounds.

## Supplementary Information

Below is the link to the electronic supplementary material.

Supplementary Material 1

## Data Availability

No datasets were generated or analysed during the current study.

## Abbreviations

4-PhB: 4-phenylbutyrate
aCD8: Anti-CD8 specific antibody
ACK: Ammonium chloride potassium
ATCC: American Type Culture Collection
B16-F10: B16
B6: C57BL/6NRj
Balb/c: Balb/cJRj
CALR: Calreticulin
DC: Dentritic cells
DMEM: Dulbeccos Modified Eagle Medium
DMEM/F12: Dulbecco’s Modified Eagle Medium/Nutrient Mixture F-12
EDTA: Ethylenediaminetetraacetic acid
ER: Endoplasmic reticulum
F/T: Freeze/thaw
FASL: FAS ligand
FDR: False discovery rate
GO: Gene Ontology
GSEA: Gene set enrichment analysis
HMGB1: High-mobility group box 1
i.p.: Intraperitoneal injection
ICD: Immunogenic cell death
MEME: Minimum Essential Medium Eagle
MFI: Mean fluorescence intensities
MTT: 3-(4,5-dimethylthiazol-2-yl)-2,5-diphenyltetrazolium bromide
NFB: Nuclear factor kappa B
p.o.: Per oral
PFA: Paraformaldehyde
PI: Propidium iodide
RPMI 1640: Roswell Park Memorial Institute 1640
s.c.: Subcutaneously
SCID: C.B.17-severe combined immunodeficiency disease
TCR: T cell receptor
TSCs: Thiosemicarbazones
