# Supplementary material for: The anticancer thiosemicarbazone triapine exerts immune-enhancing activities via immunogenic cell death induction and FAS upregulation
Source: Exp Hematol Oncol. 2025 Aug 22;14:109. doi: 10.1186/s40164-025-00700-0 (PMC12372321; doi:10.1186/s40164-025-00700-0)
Supplement: Supplementary file 1 — Supplementary Material 1 [file 40164_2025_700_MOESM1_ESM.pdf]

Supplementary material

# The anticancer thiosemicarbazone Triapine exerts immune-enhancing activities via immunogenic cell death induction and FAS upregulation

Bianca Stiller, Alessia Stefanelli, Hemma Schueffl, Marlene Mathuber, Nadiya Skorokhyd, Judith Gufler, Christine Pirker, Martin Holcmann, Rostyslav Panchuk, Maria Sibilia, Doris Marko, Walter Berger, Christian R. Kowol, Sonja Hager* and Petra Heffeter

## 1. Supplementary data

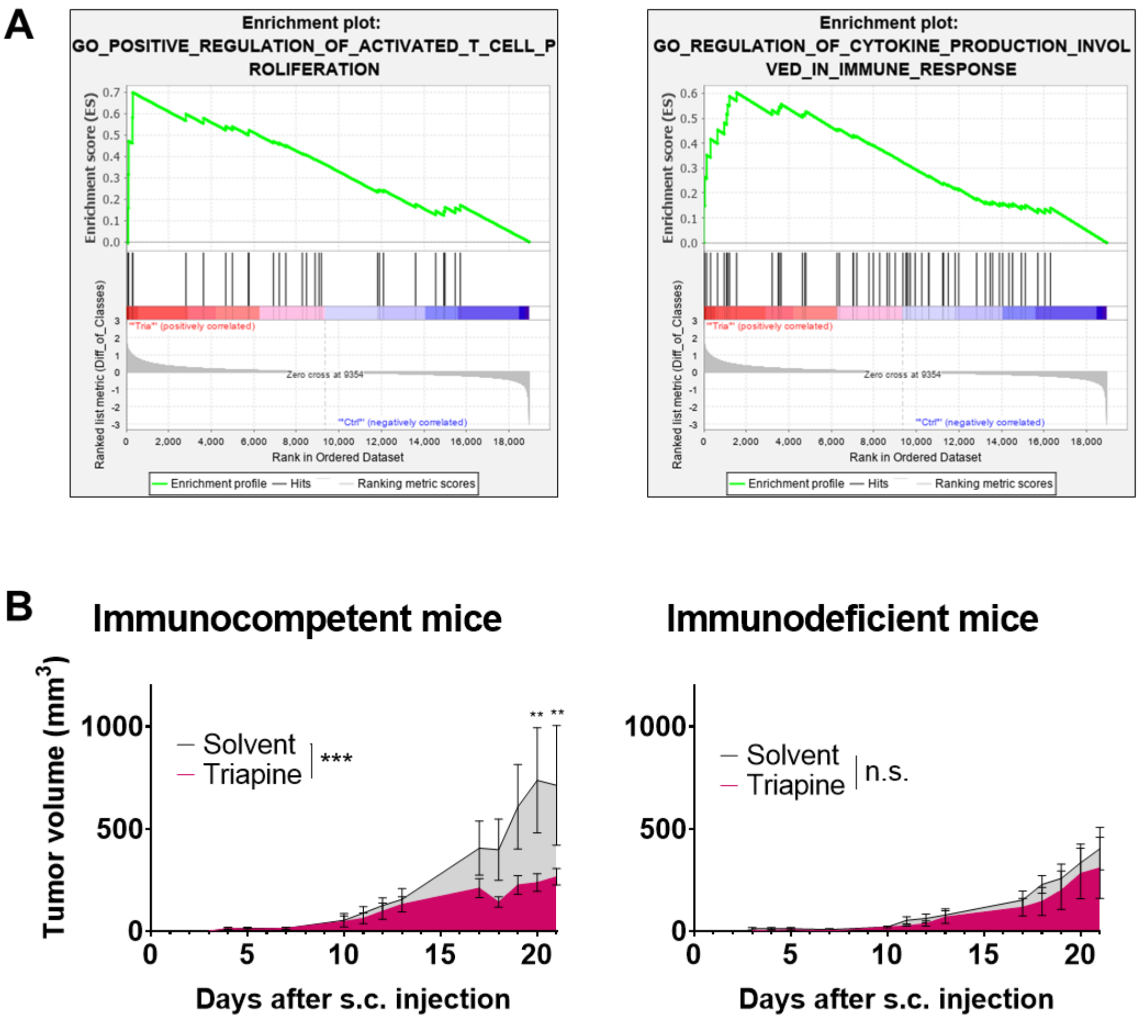


**Figure S1.** (A) Representative enrichment plots of gene set enrichment analysis (GSEA). (B) Tumor growth (B16 (n = 10)) of female C57BL/6NRj (B6) or SCID mice, treated per oral (p.o.) with Triapine (10 mg/kg) or solvent (10 % DMSO) for five consecutive days per week for two weeks. Values are mean tumor volume ($mm^3$) ± SEM. Significant differences in tumor volumes between groups were determined using mixed-effects analysis and corrected for multiple comparisons by Sidak (*** $p < 0.001$; n.s. = not significant).

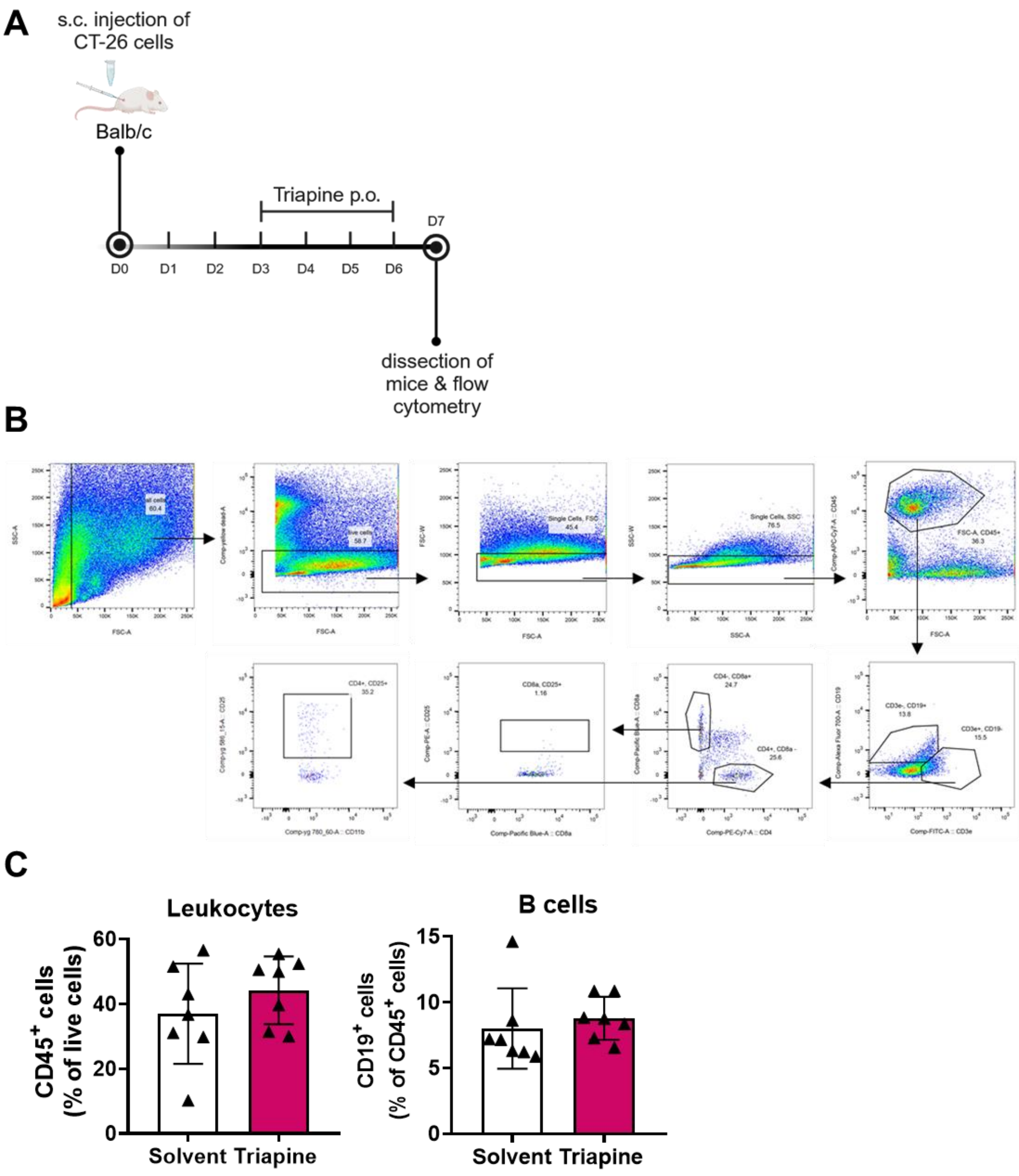


**Figure S2.** (A) Treatment scheme used for flow cytometry analysis to determine the composition of CT-26 tumor-infiltrating adaptive immune cells. of Female Balb/cJRj (Balb/c) mice (n = 7) were treated p.o. with Triapine (10 mg/kg) or solvent (10 % DMSO) for four days. On day seven multi-color flow cytometry analysis of the tumors with the indicated immune cell markers was performed. (B) Gating strategy adopted for analysis of CT-26-tumor infiltrating immune cells. (C) Results are given in mean percent cells of the (grand)parental gate ± SD. Significance between groups was calculated by unpaired T-test (** $p < 0.01$; * $p < 0.05$).

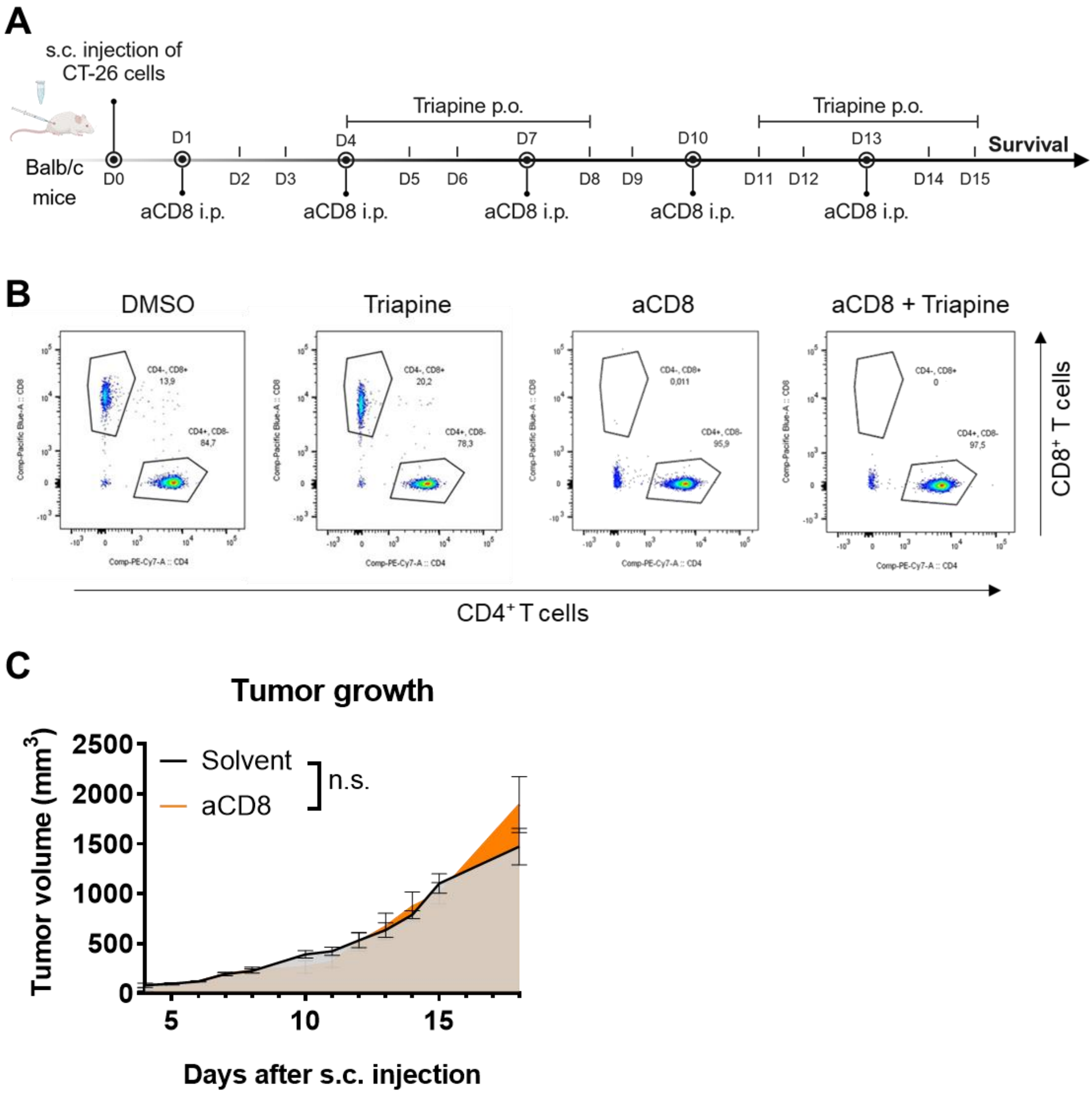


**Figure S3.** (A) Treatment scheme applied to assess the role of CD8$^+$ T cells in the activity of Triapine. Female Balb/c (n = 4) mice, treated p.o. with Triapine (10 mg/kg) or solvent (10 % DMSO) for five consecutive days per week for two weeks.In addition, mice were treated intraperitoneal injection (i.p.) with anti-CD8 specific (aCD8) antibodies (25 mg/kg) or solvent (PBS), as indicated in the scheme.(B) Flow cytometry confirmation of depletion of CD8+ T cells after treatment with anti-CD8 specific antibody. Blood samples were taken from the facial vein once every week and the immune cells were stained with fluorescence-labeled CD45-, CD3-, CD4- and CD8-antibodies for multi-color flow cytometry analysis. (C) Values are mean tumor volume (mm$^3$) ± SEM solvent- or aCD8-treated Balb/c mice. Significance was determined by two-way ANOVA corrected for multiple comparisons by Sidak (n.s. = not significant).

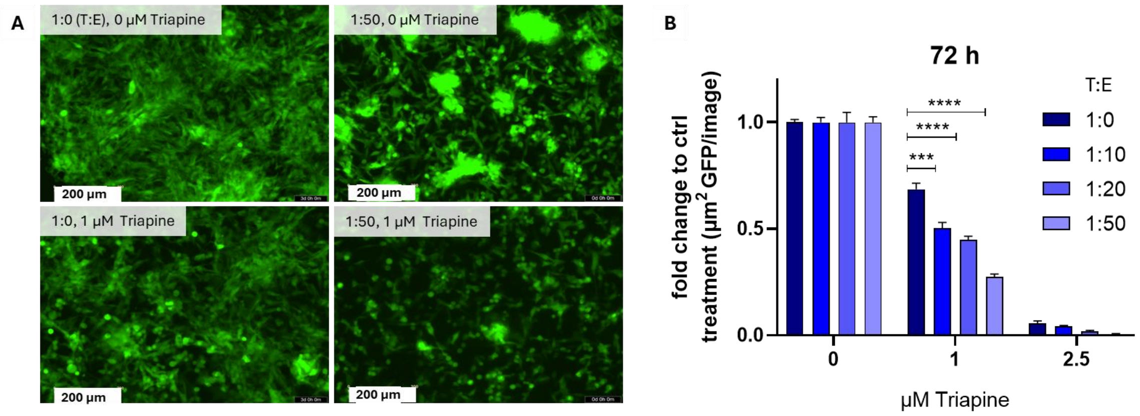


**Figure S4:** Co-culture of CD3-activated murine splenocytes with Triapine-treated CT-26 cancer cells. Cancer cells were treated with 0, 1 or 2.5 µM Triapine. After 24 h of incubation, Triapine was removed and splenocytes from Balb/c mice were added in different tumor:effector cell (T:E) ratios. The co-culture was imaged with the live-cell analysis system IncuCyte S3 after 72 h co-culture (20x objective, 300 ms exposure time). A) Representative fluorescence images are shown of (green) GFP-expressing CT-26 cells (scale bar: 200 µm). B) Quantification of fluorescence images (n = 9). The area occupied by GFP-labeled CT-26 cells was determined using the IncuCyte software (v2024). Values given are mean ± SD. Significance was calculated with two-way ANOVA and Dunnett's multiple comparison test (*** $p < 0.001$, **** $p < 0.0001$).

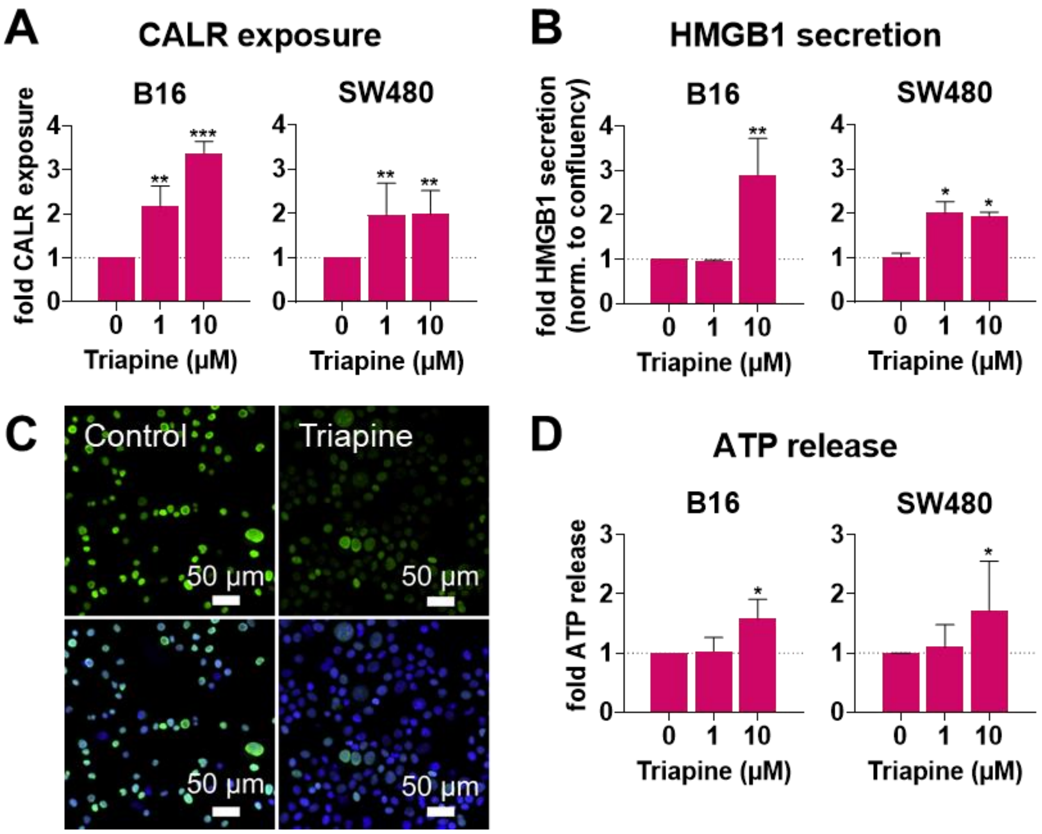


**Figure S5.** (A-D) ICD hallmarks *in vitro* in B16, SW480 and CT-26 cells, after treatment with the indicated concentrations of Triapine. (A) CALR exposure on the cell surface analyzed by flow cytometry after 24 h. (B) HMGB1 secretion was analyzed in the supernatant of treated B16 (24 h) or SW480 (48 h) cells by the Lumit™ HMGB1 Immunoassay. (C )Representative fluoresence images of CT-26 cells stained for HMGB1 (AF488) and nucleus (DAPI) after 24 h Triapine treatment (10 µM, scale bar: 50 µm). (D) ATP release was determined in the supernatant of treated cells (24 h) using the CellTiter-Glo® Luminescent Cell Viability Assay. Values given in all bar charts are the mean ± SD of at least three independent experiments, normalized to untreated control. Significance to control was determined using one-way ANOVA with Dunnett´s multiple comparisons test (*** $p < 0.001$; ** $p < 0.01$; * $p < 0.05$).

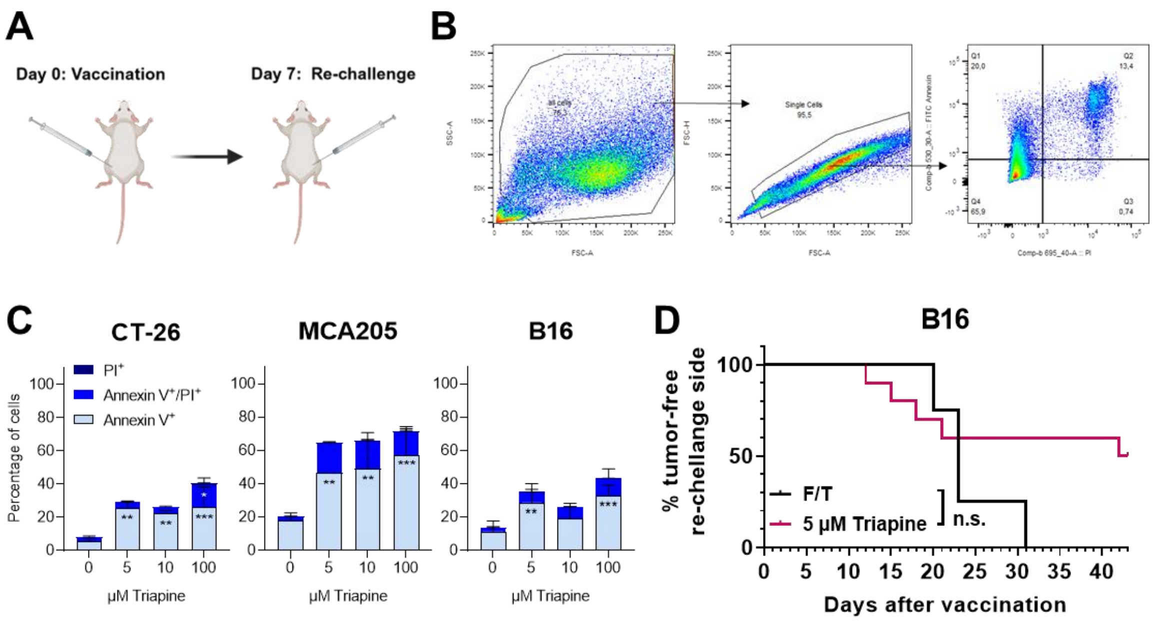


**Figure S6.** (A) Treatment scheme used for the immunization experiment with Triapine-treated cancer cells. Female Balb/c (CT-26 (n = 5)) or B6 (MCA205 (n = 5), B16 (n = 10)) mice were vaccinated with 24 h Triapine-treated CT-26, MCA205 or B16 cells by subcutaneously (s.c.) injection into the left flank on day zero. The control group received F/T-lysed cells (CT-26, MCA205 (n = 5), B16 (n = 4)). After seven days mice were re-challenged with viable CT-26 cells s.c. into the right flank, followed by monitoring of the tumor growth. (B) Gating strategy of Annexin V/ propidium iodide (PI) flow cytometry analysis of CT-26, MCA205 and B16 cells, treated with the indicated concentration of Triapine for 24 h. (C) Values given in the graph are the % of positive cells for Annexin $V^+$ or/and $PI^+$ cells of the parent gate (single cells) of two independent experiments. Significance to control within the staining groups was determined using one-way ANOVA with Dunnett´s multiple comparisons test (*** $p < 0.001$; ** $p < 0.01$). (D) Percentage of tumor-free mice at the site of re-challenge of female B6 (B16 (n = 10)) mice vaccinated with 24 h Triapine-treated cancer cells. Significant differences in median % tumor-free on site of re-challenge between groups were calculated by Log-rank test and Mantel-Cox post-test (n.s. = not significant).

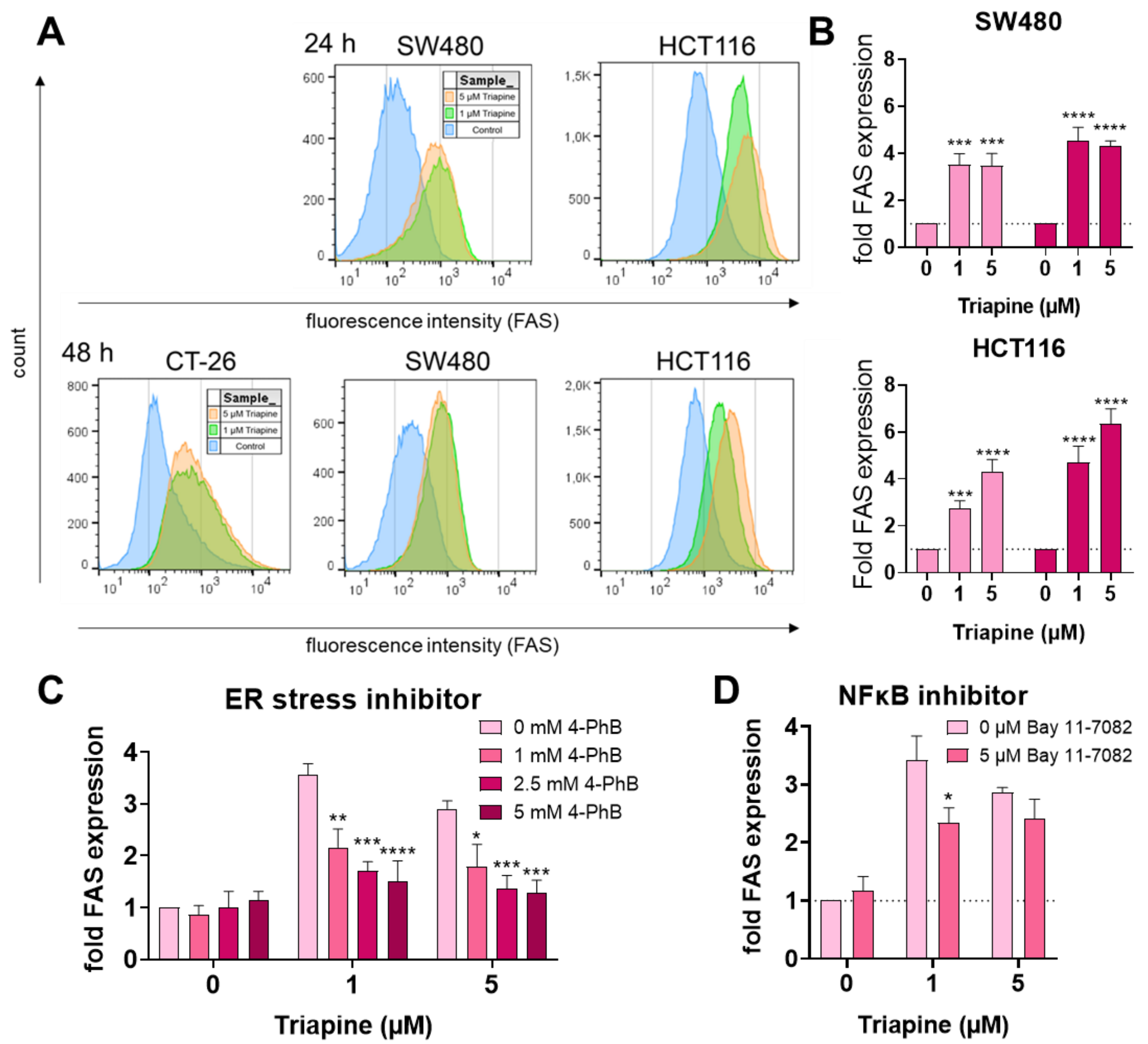


**Figure S7.** (A) Representative flow cytometry histograms and (B) quantification of FAS expression of Triapine-treated CT-26, SW480 and HCT116 cells (24 h, 48 h). Values given are the MFI ± SD of three independent experiments, normalized to control. Significance to control was determined using one-way ANOVA with Dunnett´s multiple comparisons test (**** $p < 0.0001$; *** $p < 0.001$; ** $p < 0.01$). Impact of (C) 4-PhB or ± (D) Bay 11-7082 on Triapine-induced FAS expression in CT-26 cells after 24 h treatment. Values are MFI ± SD of two independent experiments, normalized to control. Significance was determined by two-way ANOVA with Dunnett´s multiple comparisons test (**** $p < 0.001$, *** $p < 0.001$, ** $p < 0.01$, * $p < 0.05$).

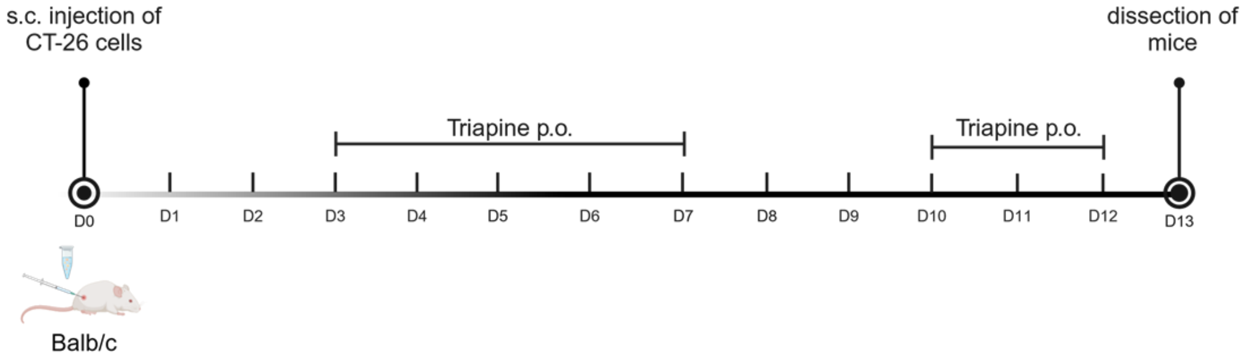


**Figure S8.** Experimental scheme of Triapine-treated (10 mg/kg) CT-26 tumor-bearing Balb/c mice shown in Figure 2 C and D. Scheme is created with BioRender.com.

## 2. Materials and methods

### 2.1. Chemicals and antibodies

Triapine (dissolved in DMSO) was purchased from BLD Pharmatech (Kaiserslautern, DE, #245946), 4-PhB (dissolved in culture medium) from MERCK (Darmstadt, Germany, #SML0309) and Bay 11-7082 (dissolved in DMSO) from Selleck Chemicals (Houston, USA, #S2913). If not mentioned separately, all other chemicals and reagents were supplied from MERCK (Darmstadt, Germany). The following antibodies were used in this study:

**Table S1:** Overview of antibodies

| Antibody | Dilution | Cat.-No. | Lot.-No. | RRID |
|---|---|---|---|---|
| **Intratumoral immunophenotyping and *in vivo* CD8+ T cell depletion:** | | | | |
| APC/Cy7 anti-mouse CD45 (30-F11) | 1:200 | bl #103116 | B311087 | AB_312981 |
| FITC anti-mouse CD3ε (145-2C11) | 1:500 | bl #100306 | B202918 | AB_312671 |
| PE/Cy7 anti-mouse CD4 (GK1.5) | 1:200 | bl #100422 | B352672 | AB_312707 |
| Brilliant Violet 421™ anti-mouse CD8a (53-6.7) | 1:100 | bl #100738 | B369854 | AB_11204079 |
| **Only in intratumoral immunophenotyping:** | | | | |
| PE anti-mouse CD25 (PC61) | 1:200 | bl #102008 | B349987 | AB_312857 |
| Alexa Fluor® 700 anti-mouse CD19 (6D5) | 1:500 | bl #115528 | B314430 | AB_493735 |

bl =Biolegend, San Diego, USA

### 2.2. Cell culture

The following human and murine cell lines and cell culture media were used:

**Table 2. Overview of cell lines and the respective cell culture media.**

| Cell line | Medium | Company |
|---|---|---|

| | | |
|---|---|---|
| murine colon carcinoma CT-26. WT (CVCL_7254) as well as its GFP-transfected subclone CT-26/GFP [1] | DMEM/F12 | ATCC, Manassas, USA, #ATCC-CRL-2638 |
| murine melanoma B16-F10 (B16), (CVCL_0159) | RPMI 1640 | Mason Research Institute, Worcester, USA |
| murine fibrosarcoma MCA205 (CVCL_VR90) | MCA205 Expansion Medium (RPMI 1640 supplemented with 2 mM glutamine, 1 mM sodium pyruvate, 1 % non-essential amino acids, 50 µM ß-mercaptoethanol) | MERCK, Darmstadt, Germany, #SCC173 |
| human colon carcinoma SW480 (sex of origin: male), (CVCL_0546) | MEME | ATCC, Manassas, USA, #CCL-228 |
| human colon carcinoma HCT116 (sex of origin: male), (CVCL_0291) | RPMI 1640 | ATCC, Manassas, USA, #CCL-247 |
| human embryonic kidney cells HEK293-Blue-hTLR7 (NF-κB/AP-1-inducible SEAP reporter gene), (CVCL_IM84) | DMEM supplemented with 2 mM L-glutamine, 100 µg/mL Normocin, 10 µg/mL Blasticidin, 100 µg/mL Zeocin, 100 units/mL of penicillin and 100 µg/mL of streptomycin | InvivoGen, Toulouse, France, #hkb-htlr7v2 |

DMEM/F12 = Dulbecco's Modified Eagle Medium/Nutrient Mixture F-12, ATCC = American Type Culture Collection, RPMI 1640 = Roswell Park Memorial Institute 1640, MEME = Minimum Essential Medium Eagle, DMEM = Dulbeccos Modified Eagle Medium

All culture media were supplemented with 10 % FBS. The cell lines were maintained under standard cell culture conditions at 37 °C in a humidified atmosphere at 5 % $CO_2$ and regularly checked for Mycoplasma contamination. Cell lines were used between passage three and twenty.

### 2.3. Whole genome gene expression analysis

Whole genome gene expression arrays and GSEA with GO terms (c5.bp.v6.0.symbols) were performed as previously described.[2] Transcriptional profiles of cells were determined by using human 4 × 44K gene expression microarrays (Agilent, Santa Clara, US, #G4845A) as described previously.[3] Normalization was performed in R using the Bioconductor (version 3.7, RRID:SCR_006442) package "limma".[4] In **Figure 2A** average of several oligonucleotide variants (if applicable) was calculated.

## 2.4. Animals

Eight- to twelve-week-old female Balb/c (IMSR_RJ:BALB-CJRJ, original source: Zentralinstitut für Versuchstierzucht (Hannover) - 1988 (F172), genotype: $yr^c/Tyr^c$, $Tyrp1^b/Tyrp1^b$, A/A - MHC: Haplotype $H2^d$, supplier: Janvier, Le Genest-Saint-Isle, France), C.B.17-SCID (IMSR_ARC:SCID, original source: Fox Chase Cancer Center, Philadelphia, Pennsylvania, genotype: IcrHan®Hsd-$Prkdc^{scid}$, supplier: Inotiv, Indiana, USA ) or B6 (IMSR_RJ:C57BL-6NRJ, original source: National Institutes of Health (USA) – 1999, genotype: a (a/a) non agouti - MHC: $HaplotypeH2^b$, supplier: Janvier, Le Genest-Saint-Isle, France) were kept in a pathogen-free environment with a 12 h light-dark cycle. Every procedure was done in a laminar airflow cabinet. Experiments were done according to the regulations of the Ethics Committee for the Care and Use of Laboratory Animals at the Medical University Vienna (proposal number 2022-0.892.227-1-A), the U.S. Public Health Service Policy on Human Care and Use of Laboratory Animals as well as the United Kingdom Coordinating Committee on Cancer Prevention Research's Guidelines for the Welfare of Animals in Experimental Neoplasia. To ensure animal welfare throughout the experiment, animals were controlled for distress development every day and body weight and tumor size was assessed regularly by caliper measurement. In the case of a tumor length >20 mm, loss of body weight (>20 % of initial weight), tumor ulceration or other indications of deteriorated health, mice were sacrificed by cervical dislocation and dissected. Tumor volume was calculated using the formula: length × $width^2$ / 2.

## 2.5. In vivo Triapine therapy in immunocompetent vs. immunodeficient mice

CT-26 ($5 \times 10^5$ cells in 50 µL RPMI 1640 without FBS (R0), n = 8), MCA205 ($5 \times 10^5$ in 100 µL R0, n = 4) or B16 ($1 \times 10^5$ in 100 µL R0, n = 12) cells were injected s.c. into the right flank of female Balb/c (CT-16), B6 (MCA205, B16) or SCID mice (CT-26, MCA205, B16). Triapine (10 mg/kg in 10 % DMSO, 200 µL/20 g mouse) or solvent treatment was given p.o. for five consecutive days a week for two (CT-26- and MCA205-bearing mice) or three (B16-bearing mice) weeks, starting on day three after cell injection. The endpoint of the experiment was overall survival.

### 2.6. Immunophenotyping of tumor-infiltrating immune cells

CT-26 (5 x $10^5$ cells in 50 µL R0, n = 7) cells were injected s.c. into the right flank of female Balb/c mice. Triapine (10 mg/kg in 10 % DMSO, 200 µL/20 g mouse) or solvent treatment was given p.o. for four days, starting on day three after cell injection. At day seven, animals were sacrificed by cervical dislocation and tumors were removed and digested in PBS ($Mg^{2+}$ and $Ca^{2+}$)/5 % FBS containing 1 mg/mL Collagenase VII (MERCK, Darmstadt, Germany, #C2139), 1 mg/mL DNAse I (MERCK, Darmstadt, Germany, #DN25) for 15 min at 37 °C, followed by straining through a 70 µm mesh (Szabo-Scandic, Vienna, Austria, #BDLBDL352350). Subsequently, erythrocytes were lysed with an ammonium chloride potassium (ACK, dd$H_2O$ with 1 M NH4Cl, 1 M KHCO3 and 1 M Titriplex III (ethylenediaminetetraacetic acid (EDTA)) buffer for 5 min at RT, stopped by adding Wuerzburg buffer (PBS with 5 % FBS, 5 mM EDTA, 20 µg/mL DNAse I). 1 x $10^6$ single cell suspensions in 50 µL Wuerzburg buffer were first blocked by using a 1 ng/mL Fc-antibody solution (clone: 93, Biolegend, San Diego, USA, #101330, Lot.-No.: B348020, RRID: AB_2561482) for 10 min and then stained for 30 min at RT with the indicated antibodies in **Figure 2C, S2C** and **Table S1**. Ten min before flow cytometry measurement, Sytox blue (1:400, Thermo Fisher Scientific, Waltham, USA, #S34857) was added to stain for dead cells. All samples were measured on a BD LSRFortessa™ X-20 cell analyzer (Becton Dickinson, Palo Alto, USA) and analyzed using FlowJo™ (FlowJo LLC, Ashland, USA, version 10, RRID: SCR_008520). The gating strategy is shown in **Figure S2B**.

### 2.7. In vivo $CD8^+$ T cell depletion

CT-26 cells (5 x $10^5$ cells in 50 µL serum-free culture medium) were injected s.c. into the right flank of female Balb/c mice (n = 16). Four groups (n = 4) were randomly formed. For achieving $CD8^+$ T cell depletion, the first and the second group were treated with anti-CD8 specific antibodies (clone 53.6.7, rat [Lou/Ws1/M] IgG2aK), 2 mg/mL in PBS, 250 µL/20 g mouse), i.p. once every three days, starting on day one after cell injection. In addition, the second group was treated with Triapine (10 mg/kg in 10 % DMSO, 200 µL/20 g mouse) p.o. for five consecutive days per week for two weeks, starting on day three after cell injection. Group one was additionally treated with solvent instead of Triapine. As control groups, the third group was treated with Triapine and PBS instead of antibody and the fourth group received only solvents. The endpoint of the experiment was overall survival. To confirm depletion of $CD8^+$ T cells,

blood samples were taken from the facial vein once every week (by using heparin-coated tubes to prevent coagulation). Subsequently, erythrocytes were lysed, followed by blocking, staining for immune marker (used antibodies indicated in **Table S1**) as well as dead cells, measuring by flow cytometry and quantified as mentioned in **section 2.6**.

### 2.8. CALR exposure analysis by flow cytometry

Cells were seeded (3 x $10^5$ cells well) in 6-well plates and allowed to recover for 24 h. After the indicated treatments, cells were trypsinized and incubated for 1 h with the monoclonal rabbit anti-CALR (D3E6) antibody (1:800, Cell Signaling Technology, Beverly, USA, #12238, Lot.-No.: 4, RRID: AB_2688013) in 0.5 % BSA in PBS on ice. Subsequently, the cells were incubated for 30 min on ice with the secondary AF488-conjugated anti-rabbit antibody (1:200 in 0.5% BSA in PBS, Thermo Fisher Scientific, Waltham, USA, #A11034, Lot.-No.: 2110499, RRID: AB_2576217). Finally, the cells were measured and analyzed as mentioned in **section 2.6.**

### 2.9. ATP release assay

Cells (5 x $10^3$ cells/well) were plated into 96-well plates and left to recover for 24 or 48 h. Treatment was performed as indicated and all supernatants collected. Dying tumor cells or cell debris were removed by centrifugation (5 min, 300 x g). The supernatants were transferred into a solid white tissue culture plate (Greiner Bio-One, Kremsmuenster, Austria, #655073) and the ATP levels were measured using the CellTiter-Glo kit according to the manufacturer's instructions (Promega, Madison, USA, #G9681) by Infinite ® 200 PRO plate reader (integration time: 1000 ms, settle time: 0 ms, Tecan Trading AG, Maennedorf, Swizerland). Outliers were excluded according to Grubbs' test in GraphPad Prism (RRID: SCR_002798).

### 2.10. HMGB1 secretion assay

Cells (5 x $10^3$ cells/well) were plated into 96-well plates and left to recover for at least 24 h. Treatment was performed as indicated and 80 µL of the supernatant were transferred into a solid white tissue culture plate (Greiner Bio-One, Kremsmuenster, Austria, #655073). The Lumit™ HMGB1 (Human/Mouse) Immunoassay was purchased from Promega, Madison, USA (#W6110) and performed according to the manufacturer's instructions. Luminescence has been detected by Spark® multimode

microplate reader (integration time: 1000 ms, settle time: 0 ms, Tecan Trading AG, Maennedorf, Swizerland).

## 2.11. CALR immunofluorescence

$10^5$ cells/mL (200 µl/well) were seeded onto ibidi slides (coated with polymers, Science Services, #80806) and allowed to recover at 37 °C with 5 % $CO_2$ o.n. The next day, the cells were treated with the indicated concentration of Triapine at 37 °C with 5 % $CO_2$ for 24 h. Subsequently, cells were fixed with 0.25 % paraformaldehyde (PFA) for 10 min at 4 °C, followed by washing with PBS and staining with a monoclonal antibody specific for CALR (D3E6, rabbit, CS, #12238, Lot.-No.: 4, RRID: AB_2688013) 1:200 in 20 % FBS in PBS for 1.5 h at RT. Subsequently, the cells were incubated with the anti-rabbit Alexa Flour 488 labeled-secondary antibody (1:200 in 20 % FBS in PBS, Thermo Fisher Scientific, #A-11034, Lot.-No.: 2110499, RRID: AB_2576217) for 30 min at RT and an additional fixation step with 4 % PFA for 30 min at 4 °C. After three PBS washing steps, the cells were stained with DAPI (2.5 µg/mL in PBS) for 15 min at RT, followed by embedding with Vectashield (Vectashield® Antifade Mounting Media, Vector Laboratories #H-1000-10). In case of CALR, confocal microscopy was performed on a Zeiss LSM 700 (Carl Zeiss AG) equipped with 405 and 488 nm solid state laser diodes, using a Plan-NeoFluar 40x/NA 1.3/oil objective. The pinhole size was set to one AU. Line average of two was applied to all channels. In total, three pictures per well were taken.

## 2.12. HMGB1 immunofluorescence

3 x $10^5$ cells/mL (1 mL/well) were seeded on 6 well plates and allowed to recover at 37 °C with 5 % $CO_2$ o.n. The next day, the cells were treated with the indicated concentration of Triapine at 37 °C with 5 % $CO_2$ for 24 h. Subsequently, cells were trypsinized and transferred to microscopy slides using a cytocentrifuge (Cytospin 4, Thermo Scientific, USA) (100 g, 8 min). Next, cells were left to dry fo 30 min, followed by fixation with aceton:methanol (1:1) for 10 min at 4 °C. After washing, cells were blocked and permeabilized with 5 % FBS and 0.3 % Triton X-100 in PBS for 1 h at RT, stained with a polyclonal antibody specific for HMGB1 (rabbit, CS, #3935, Lot.-No.: 2, RRID: AB_2295241) 1:50 in 3 % FBS and 0.3 % Triton X-100 in PBS o.n. at 4 °C, followed by incubation with the anti-rabbit Alexa Flour 488 labeled-secondary antibody as mentioned above. After three PBS washing steps, the cells were stained with DAPI

(2.5 µg/mL in PBS) for 15 min at RT, followed by embedding with Vectashield (Vectashield® Antifade Mounting Media, Vector Laboratories #H-1000-10). Fluorescence microscopy was performed on an Olympus iX83, using a Univ.C-Planfluorit 40x/NA 0.6/air objective (exposure time: Alexa Flour 488 = 100 ms, DAPI = 1 ms). In total, 4-5 pictures per well were taken. HMGB1 signal (AF488) was measured within the nucleus (DAPI) by the quantification software (CellSens Dimension V4.4, Evident Scientific)

**2.13. Annexin V-/PI-staining**

3 x $10^5$ cells/well were seeded into 6-well plates. After o.n. recovery, the cells were treated for 24 h with the indicated drug concentrations. Subsequently, the supernatant and the cells were collected and stained with Annexin V-FITC (1:50, BD Biosciences, New Jersey, USA, #556420) and PI (1 µg/mL MERCK, Darmstadt, Germany, #P4864) in Annexin V-binding buffer (10 mM HEPES, 140 mM NaCl, 2.5 mM $CaCl_2$x2 $ddH_2O$) for 15 min at RT. Fluorescence intensity was measuring by flow cytometry and quantified as mentioned in **section 2.6**.

**2.14. Immunization**

3 x $10^6$ cells were seeded in T75 flasks and left to recover for 24 h. Treatment was administered as indicated and cells were collected by trypsinization (including dead cells in growth medium). After washing, 1 x $10^6$ cells in 100 µL PBS (without $Ca^{2+}$, $Mg^{2+}$) were injected s.c. into the left flank of mice for vaccination. As a negative control, untreated cells subjected to repeated freeze/thaw (F/T) cycles for cell fragmentation without induction of immunogenic signals were used. After seven days, the same cells, without treatment, were injected (5 x $10^5$ in 100 µL PBS without $Ca^{2+}$, $Mg^{2+}$) s.c. into the right flank of mice. Tumor growth was measured every day on both injection sites. The endpoint of the experiment was overall survival.

**2.15. Co-culture of cancer cells with splenocytes**

8 x $10^3$ CT-26/GFP per well were seeded in 24-well plates in phenol red-free growth medium and left to recover. On the next day, the cells were treated with 0, 1 or 2.5 µM Triapine for 24 h. Splenocytes were isolated from spleens of healthy female Balb/c mice by straining the tissue through a 70 µm mesh to get a single cell suspension, followed by lysis of erythrocytes by ACK buffer for 5 mins at RT. Then, splenocytes

were incubated on an anti-CD3 antibodies (Invitrogen, #16-0031-82)-coated plate in RPMI 1640 medium with 10% FBS and 50 µM ß-Mercaptoethanol overnight (o.n.). Subsequently, splenocytes were added to the CT-26/GFP in target to effector cell (T:E) ratio of 1:0, 1:20 and 1:50 for 72 h. Cancer cell viability was measured and quantified by the GFP-positive area of the cancer cells (Ex: 469/35, Em: 525/39) of the whole well by the fully equipped monochromatic based plate reader IncuCyte S3 (Sartorius) with a 20x phase-contrast objective. During imaging the cells were incubated in a heated chamber warmed to 37 °C. The GFP-positive area of the CT-26/GFP cells were quantified by IncuCyte software (version 2024).

**2.16. FAS expression analysis by flow cytometry**

CT-26, SW480 (2 x $10^5$ cells/well for 24 h, 1 x $10^5$ cells/well for 48 h) or HCT116 cells (5 x $10^5$ cells/well for 24 h, 2.5 x $10^5$ cells/well for 48 h) were seeded into 6-well plates and allowed to recover for 24 h. After incubation with the indicated drugs for 24 or 48 h, cells were trypsinized and incubated with the FVD780 stain (Thermo Fisher Scientific, Waltham, USA, #65-2860-40) for 15 min on ice. In case of the CT-26 model, cells were blocked as mentioned in section 4.6. Subsequently, CT-26 cells were incubated with the PE/Dazzle™ 594 anti-mouse CD95 (Fas) antibody (SA367H8, 1:200, Biolegend, San Diego, USA, #152626, Lot.-No.: B404978, RRID: AB_3068247) and SW480 as well as HCT116 cells with the Brilliant Violet 711™ anti-human CD95 (Fas) antibody (DX2, 1:200, Biolegend, San Diego, USA, #305644, Lot.-No.: B384921, RRID:AB_2632623) for 30 min at RT. Finally, the cells were measured and quantified as mentioned in **section 2.6**.

**2.17. Immunohistochemistry**

CT-26 (5 x $10^5$ cells in 50 µL R0, n = 4) cells were injected s.c. into the right flank of female Balb/c. Triapine (10 mg/kg in 10 % DMSO, 200 µL/20 g mouse) or solvent treatment was given as indicated in scheme **Figure S7**. On the day 13 the animals were sacrificed by cervical dislocation and tumors collected for fixation in 4 % formaldehyde for 24 h (Carl Roth, Karlsruhe, Germany, #P087.4) followed by paraffin embedding using a KOS machine (Milestone, Valbrembo, Italy). For histological evaluation, tumor tissues were sliced in 4 µm thick sections. Fresh sections were deparaffinized and dehydrated. After antigen retrieval by boiling for 30 min in 10 mM citrate buffer (pH 6.0), sections were incubated with anti-FAS antibody (1:200, Thermo

Fisher Scientific, Waltham, USA, #PA5-115214, Lot.-No.: YJ4106279, RRID:AB_2899850) in a humid chamber for 1 h at RT. Antibody binding was detected using the UltraVision LP detection system according to the manufacturer's instructions (Thermo Fisher Scientific, Waltham, USA, #TL-125-HD). Scanning of the slides was performed with a 3D Histech Microscopic High Throughput slidescanner for Brightfield (Pannoramic SCAN II Scanner, 3DHistech, Budapest, Hungary). Evaluation and quantification of the staining were done by Halo software (Indica Labs, Albuquerque, USA, version 3.6). Important to note, one mouse had such a small tumor after Triapine treatment, that no IHC staining was possible. objective: 20x,

**2.18. Cell viability assay**

HCT116 cells (3 x $10^3$ cells/well) were plated into 96-well plates and allowed to recover for 24 h. Subsequently, cells were treated with the indicated concentration of Triapine for 24 h followed by an additional treatment with soluble FASL (Thermo Fisher Scientific, Waltham, USA, #310-03H) for further 24 h. Cell viability was measured by the 3-(4,5-dimethylthiazol-2-yl)-2,5-diphenyltetrazolium bromide (MTT)-based vitality assay (EZ4U, Biomedica, Vienna, Austria, #BI-5000) according to the manufacturer's instructions.

**2.19. Caspase-Glo® 8 assay**

HCT116 cells (3 x $10^3$ cells/well) were seeded in white/clear 96-well plates (Falcon®, VWR International LLC., Vienna, Austria, #353377). On the next day, cells were treated with the indicated concentration of Triapine for 24 h followed by an additional treatment of soluble FASL (Thermo Fisher Scientific, Waltham, USA, #310-03H) for further 24 h. Caspase 8 activity was measured by the Caspase-Glo® 8 assay (Promega, Madison, USA, #G8200) according to the manufacturer's instructions. Luminescence was detected by Spark® multimode microplate reader (integration time: 1000 ms, settle time: 0 ms, Tecan Trading AG, Maennedorf, Swizerland).

**2.20. NFκB activity assay**

HEK-Blue-hTLR7 (5 x $10^5$ cells/mL) were seeded in 96-well plates, incubated o.n., followed by a Triapine treatment with the indicated concentration for 24 h. Subsequently, 20 µL of the cell supernatant were transferred to new 96-well plates, 180 µL of the QB solution (InvivoGen, Toulouse, France, #rep-qbs3) were added and

incubation performed for 2 h at 37 °C. SEAP levels were detected colorimetrically by Spark® multimode microplate reader (absorbance: 620 nm, Tecan Trading AG, Maennedorf, Swizerland).

### 2.21. Statistical analysis

All statistical analyses were performed and graphs were created using GraphPad Prism v8.0.1 (Boston, USA, RRID: SCR_002798). Statistical significance was evaluated using unpaired two-tailed student's t test, one-way, two-way ANOVA with multiple comparison test by Bonferroni or Dunnett, mixed-effects analysis with multiple comparisons by Sidak, Log-rank test and Mantel-Cox post-test as indicated in the Figure legends. P-values $< 0.05$ were considered statistically significant p.

### 2.22. Data Availability Statement

The data generated in this study are available upon request from the corresponding author.

## Supplementary References


1. Groza D, Gehrig S, Kudela P, Holcmann M, Pirker C, Dinhof C, et al. Bacterial ghosts as adjuvant to oxaliplatin chemotherapy in colorectal carcinomatosis. Oncoimmunology. 2018;7(5):e1424676.
2. Hager S, Korbula K, Bielec B, Grusch M, Pirker C, Schosserer M, et al. The thiosemicarbazone Me2NNMe2 induces paraptosis by disrupting the ER thiol redox homeostasis based on protein disulfide isomerase inhibition. Cell death & disease. 2018;9(11):1052.
3. Laszlo V, Hoda MA, Garay T, Pirker C, Ghanim B, Klikovits T, et al. Epigenetic down-regulation of integrin alpha7 increases migratory potential and confers poor prognosis in malignant pleural mesothelioma. The Journal of pathology. 2015;237(2):203-14.
4. Ritchie ME, Phipson B, Wu D, Hu Y, Law CW, Shi W, et al. limma powers differential expression analyses for RNA-sequencing and microarray studies. Nucleic acids research. 2015;43(7):e47.
